# Down-regulation of long non-coding RNA MEG3 suppresses osteogenic differentiation of periodontal ligament stem cells (PDLSCs) through miR-27a-3p/IGF1 axis in periodontitis

**DOI:** 10.18632/aging.102105

**Published:** 2019-08-09

**Authors:** Yi Liu, Chunpeng Liu, Ankui Zhang, Shichang Yin, Ting Wang, Yan Wang, Meiming Wang, Yixin Liu, Qiaohui Ying, Jinrui Sun, Fulan Wei, Dongxu Liu, Chunling Wang, Shaohua Ge

**Affiliations:** 1 Shandong Provincial Key Laboratory of Oral Tissue Regeneration, School of Stomatology, Shandong University, Jinan, Shandong 250012, China; 2 Department of Orthodontics, School of Stomatology, Shandong University, Jinan, Shandong 250012, China; 3 Department of General Dentistry, School of Stomatology, Shandong University, Jinan, Shandong 250012, China; 4 Department of Periodontology, School of Stomatology, Shandong University, Jinan, Shandong 250012, China

**Keywords:** osteogenic differentiation, MEG3, miR-27a-3p, *IGF1*, PDLSCs

## Abstract

Objective: This study aimed to investigate the roles of long noncoding RNA (lncRNA) maternally expressed gene 3 (MEG3) in osteogenic differentiation of periodontal ligament stem cells (PDLSCs) in periodontitis.

Methods: Differentially expressed lncRNAs and mRNAs between periodontitis periodontal ligament tissues and healthy periodontal ligament tissues were selected out using R project. PDLSCs were identified using flow cytometry. Western blot was employed to detect pathway relative proteins. Besides, targeted relationships between lncRNA and miRNA, as well as miRNA and mRNA were verified by dual luciferase reporter gene assay. Osteogenic differentiation of PDLSCs was assessed by alkaline phosphatase (ALP) staining and Alizarin Red Staining (ARS). Markers for osteoblast (Runx2, Osterix, Osteocalcin, Colla1) were detected using western blot.

Results: LncRNA MEG3 and *IGF1* were both down-regulated, while miR-27a-3p was up-regulated in periodontitis samples compared with healthy samples. Overexpression of MEG3 promoted osteogenic differentiation by enhancing expression of *IGF1* yet suppressing expression of miRNA-27a-3p. Meanwhile, the results of ALP and ARS staining indicated that up-regulation of lncRNA MEG3 or *IGF1* promoted osteogenic differentiation in PDLSCs, which could be reversed with up-regulation of miRNA-27a-3p.

Conclusion: Down-regulation of MEG3 suppressed osteogenic differentiation of PDLSCs through miR-27a-3p/*IGF1* axis in periodontitis.

## INTRODUCTION

Periodontitis is a widespread infectious human chronic inflammatory disease which is characterized by progressive disruption of the connective tissue surrounding the teeth and also the inflammatory reaction mostly caused by an oral microbial biofilm [1]. Globally, serious periodontitis infects 9% to 11% of adult population in the world, especially in adult population over 50 years of age [2–4]. If untreated, periodontitis will induce the destruction of the periodontal structures, including alveolar bone, periodontal ligament, and root cementum, which will eventually lead to tooth-loss [5]. Therefore, investigating the pathogenesis of periodontitis is extremely important in the prevention and remedy of the disease.

Periodontal ligament stem cells (PDLSCs) are a group of mesenchymal stem cells (MSCs) derived from the periodontal ligament, which show multiple differentiation capability and a promising potential of being used to regenerate supporting tissues for the patients recently [6, 7]. Current researches have demonstrated that activating phosphatidylinositol 3-kinase (PI3K) / Akt signaling pathway could regulate the osteogenic differentiation of PDLSCs [8]. Though most of the studies focus on cancers, there are still some of the researches for periodontitis [9–11].

LncRNAs, a class of RNAs that are >200 bases in length, have recently drawn wide attention in describing the complex mechanisms underlying malignant processes, such as carcinogenesis and periodontitis. LncRNAs have been reported to engage in numerous biological processes, such as transcription, post-transcription, and translational regulation of gene expression [12]. For example, PTENP1, a pseudogene transcript of the tumor-inhibiting factor phosphatase and tensin homolog (PTEN), was targeted by endogenous miRNA, and quenched the endogenous miRNAs [13]. Though many current studies of lncRNAs focus on cancer, some researches have reported roles in lncRNAs in periodontitis [9–11]. LncRNAs were indicated by microarray analysis to exert partial or crucial roles in periodontitis pathogenesis [14]. For one, previous study had demonstrated that lncRNA ANCR played a vital role in regulating the proliferation and osteogenic differentiation of PDLSCs [15, 16]. In addition, H19, another important lncRNA, was reported to play important roles in osteogenic differentiation of PDLSCs via wnt/β-cantein signaling pathway [17]. In brief, studies mentioned above have tentatively shed light on the roles of lncRNAs in osteogenic differentiation of PDLSCs and our study will further investigate the effects of lncRNA MEG3 in osteogenic differentiation of PDLSCs via PI3K/Akt signaling pathway.

Furthermore, a new regulation in which lncRNAs can cross-talk with mRNAs through competition for shared miRNA-response elements has been certified recently. In this circumstance, lncRNAs function as ceRNAs which means miRNA sponges or antagomirs, to affect the expression levels and activities of miRNAs, thereby repressing miRNA targets and causing an additional level of posttranscriptional regulation [18]. The overall activity and functional balance of gene networks was maintained by the lncRNA/miRNA/mRNA regulatory interactions, and any destabilization of this system may lead to pathological changes [19], and the lncRNA POIR/miR-182 regulatory network was found to play significant roles in osteogenic differentiation of PDLSCs in periodontitis [20]. Hence, a fresh perspective would be produced to research the complex mechanism underlying lncRNA/miRNA/mRNA in PDLSCs.

In this study, microarray and KEGG analysis helped identify differentially expressed lncRNA MEG3 and mRNA IGF1 in PDLSCs, as well as a regulated PI3K/Akt pathway. As co-expression networks predicted correlation between LncRNA MEG3 and *IGF1*, and between *IGF1* and PI3K/Akt related miRNAs were screened and miR-27a-3p stood out as a possible bridge between lncRNA MEG3 and *IGF1*. Besides, we determined the levels of lncRNA MEG3, miR-27a-3p and *IGF1* in PDLSCs, and investigated the cross-talk on osteogenic differentiation of PDLSCs. In addition, mechanism analysis revealed that lncRNA MEG3 may serve as a ceRNA to regulate the expression of *IGF1* by competing for miR-27a-3p, thus effecting osteogenic differentiation via PI3K/Akt signaling pathway. These findings shed new light on the diagnosis and therapy for periodontitis. Above all, we proposed that there was an association between osteogenic differentiation of PDLSCs and lncRNA MEG3.

## RESULTS

### *IGF1* was down-regulated and PI3K/Akt signaling pathway was dys-regulated in periodontitis periodontal tissues compared with healthy periodontal tissues

**Figure 1:**
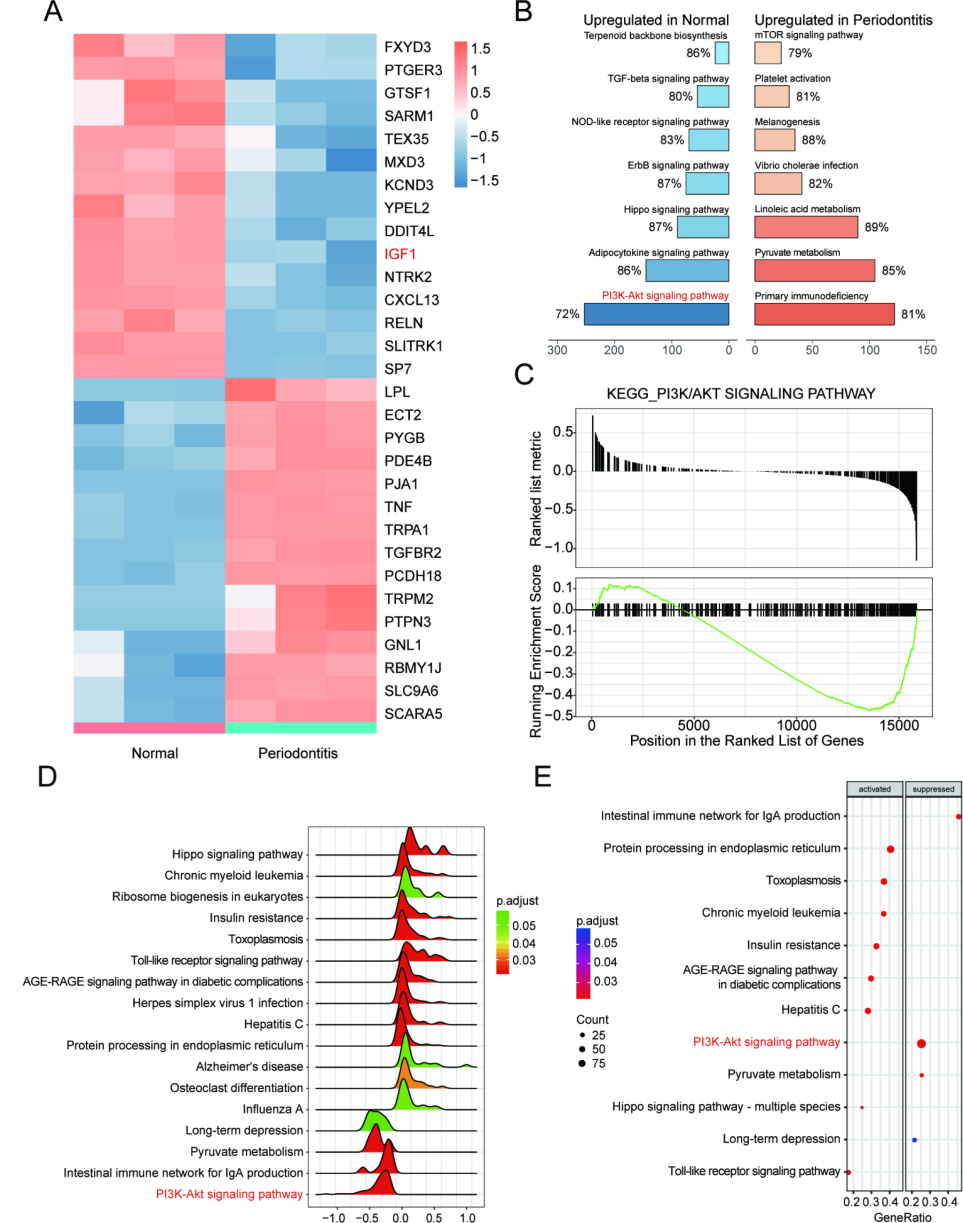
***IGF1* was down-regulated and PI3K/Akt signaling pathway was dys-regulated in periodontitis periodontal tissues compared with healthy periodontal tissues.** (**A**) Heatmap of top 15 up-regulated or down-regulated mRNAs between periodontitis periodontal tissues and healthy periodontal tissues. (**B**, **C**) KEGG pathway enrichment analysis using GSEA. PI3K/Akt signaling pathway was one of pathways that were inactivated in periodontal tissues with periodontitis compared with healthy samples. (**D**–**E**) Joyplot and dotplot of abnormally regulated signaling pathway in periodontitis tissues compared with the that in healthy samples.

Bioinformatic differential analysis selected out significantly differentially expressed genes (DEGs) between periodontitis periodontal tissues and healthy periodontal tissues. Top 15 up-regulated and down-regulated mRNAs were visualized by heatmaps as shown in Figure 1A. Thereafter, KEGG pathway enrichment analysis using gene set enrichment analysis (GSEA) software suggested that PI3K/Akt signaling pathway was inactivated in periodontal tissues with periodontitis compared with healthy samples (Figure 1B, 1C), which was better visualized by joyplot and dotplot as shown in Figure 1D–1F. Taken together, *IGF1* down-regulated in PI3K/Akt signaling pathway could be inactivated in periodontitis periodontal tissues compared with healthy periodontal tissues.

### LncRNA MEG3 was down-regulated in periodontitis periodontal tissues compared with healthy periodontal tissues and correlated with *IGF1* via miR-27a-3p

**Figure 2:**
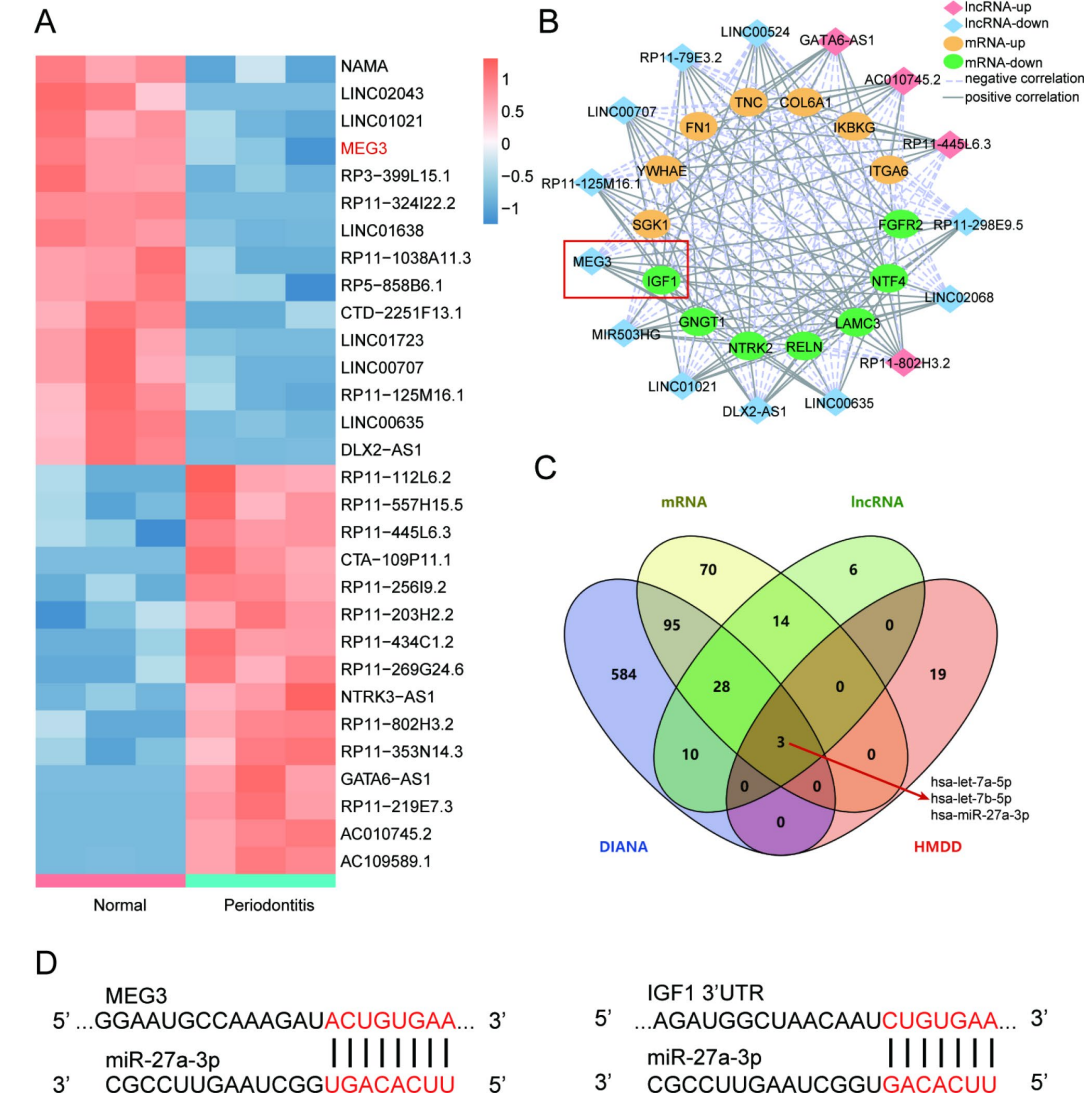
**LncRNA MEG3 was down-regulated in periodontitis periodontal tissues compared with healthy periodontal tissues and correlated with IGF1 via miR-27a-3p.** (**A**) Heatmap of top 15 up-regulated or down-regulated lncRNAs between periodontitis periodontal tissues and healthy periodontal tissues. (**B**) Co-expression network between differentially expressed lncRNAs and mRNAs in R software and Cytoscape. Screening conditions was Pearson Correlation Coefficient>0.75 and P<0.05. LncRNA MEG3 was positively correlated to *IGF1*. (**C**) 3 miRNAs directly targeting MEG3 and *IGF1* were screened by DIANA Tools and HMDD analysis, including miR-27a-3p. (**D**) Predicted binding sites of miR-27a-3p targeting MEG3 and *IGF1*.

Results of differential analysis displayed top 15 up-regulated and down-regulated lncRNAs that were significantly differentially expressed between periodontitis periodontal tissues and healthy periodontal tissues (Figure 2A). R software and Cytoscape were employed to build a co-expression network between differentially expressed lncRNAs and mRNAs according to screening conditions including Pearson Correlation Coefficient>0.75 and *P*<0.05. The results indicated that lncRNA MEG3 had positive correlation with *IGF1* (Figure 2B). Furthermore, qRT-PCR helped verify the expression of the top-5 dys-regulated lncRNAs in healthy and periodontitis PDLSCs, and detected the influences caused by osteogenic injection. Thereinto MEG3 displayed the most remarkable difference (Supplementary Figure 1A, 1B) and we chose it in our further studies. According to the results of DIANA Tools and HMDD analysis, 3 miRNAs directly targeting MEG3 and *IGF1* were screened (Figure 2C). In the present study, we focused on miR-27a-3p and the predicted binding sites with MEG3 and IGF1 were shown in Figure 2D.

### Establishment of co-expression network and identification of PDLSCs and lncRNA MEG3 was positively related with PDLSC osteogenic differentiation

**Figure 3:**
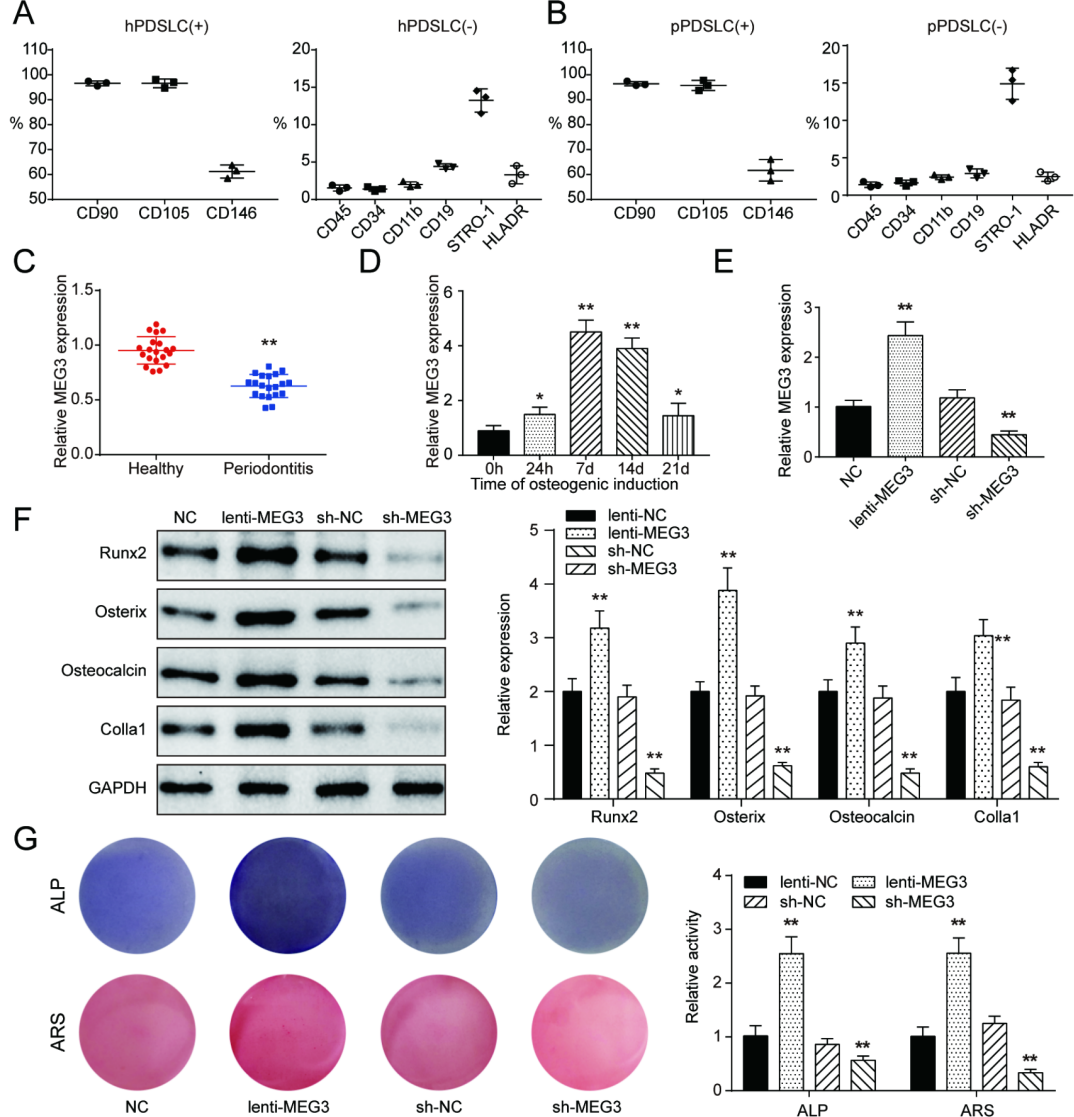
**Establishment of co-expression network and identification of PDLSCs and lncRNA MEG3 was positively related with PDLSC osteogenic differentiation.** (**A**) Expression of hPDLSC surface antigens including CD90, CD105, CD146, CD45, CD34, CD11b, CD19, STRO-1, and HLADR. (**B**) Expression of pPDLSC surface antigens including CD90, CD105, CD146, CD45, CD34, CD11b, CD19, STRO-1, and HLADR. (**C**) MEG3 was down-regulated in periodontitis tissues compared with heathy samples. (**D**) Expression of MEG3 mRNA in 0h, 24h, 7d, 14d and 21d after osteogenic induction. (**E**) Expression of MEG3 mRNA in PDLSCs treated with or without lenti-MEG3, sh-NC or sh-MEG3. (**F**) Western blot of osteoblast makers including Runx2, Osterix, Osteocalcin and Colla1. (**G**) ALP and Alizarin Red staining of PDLSCs treated with or without lenti-MEG3, sh-NC or sh-MEG3. ** *P* < 0.05, * *P* < 0.01, compared with the NC group.

To confirm whether *IGF1* could regulate PI3K/Akt signaling pathway by interacting with genes in PI3K/Akt signaling pathway, PDLSCs were isolated for following assays. Based on the results of bioinformatic analysis, following experiments were built based on lncRNA MEG3/miR-27a-3p/*IGF1* axis in this study. In addition, PDLSC surface antigens including CD90, CD105, CD146, CD45, CD34, CD11b, CD19, STRO-1, and HLADR were identified by flow cytometry and the results were demonstrated in Figure 3A, 3B and Supplementary Figure 2A, 2B. Results showed that PDSLC were positive for CD90, CD105, and CD146, and negative for CD45, CD34, CD11b, CD19, STRO-1, and HLADR. Comparison between periodontitis tissues and heathy samples indicated that expression of lncRNA MEG3 was significantly down-regulated in pPDLSCs compared with in hPDLSCs (Figure 3C). Furthermore, we detected the expression of MEG3 in pPDLSCs of older donors (age > 60, number = 10), results showed that expression of MEG3 was lower in older donors than in young donors (Supplementary Figure 3A). In osteogenic induction for 24 hours and 7, 14, 21 days, lncRNA MEG3 gained its highest expression in the 7-days induction group (Figure 3D). LncRNA MEG3 was significantly up-regulated in osteogenic PDLSCs compared with control group. Besides, after transfection of lenti-MEG3 or sh-MEG3, expression of MEG3 was respectively up-regulated or down-regulated, indicating successful transfection (Figure 3E). Expression levels of osteoblast makers, including Runx2, Osterix, Osteocalcin and Colla1 were examined with western Blot, which revealed to rise in the group of MEG3 overexpression, and to decline in the group of MEG3 knockdown (Figure 3F). ALP and Alizarin Red staining showed parallel results that osteogenic activities of PDSLCs could be promoted by overexpression of lncRNA MEG3, or repressed by silencing of lncRNA MEG3 (Figure 3G). In brief, all these results revealed that lncRNA MEG3 was positively related with PDLSC osteogenic differentiation.

### LncRNA MEG3 regulated PDLSC osteogenic differentiation through miR-27a-3p

**Figure 4:**
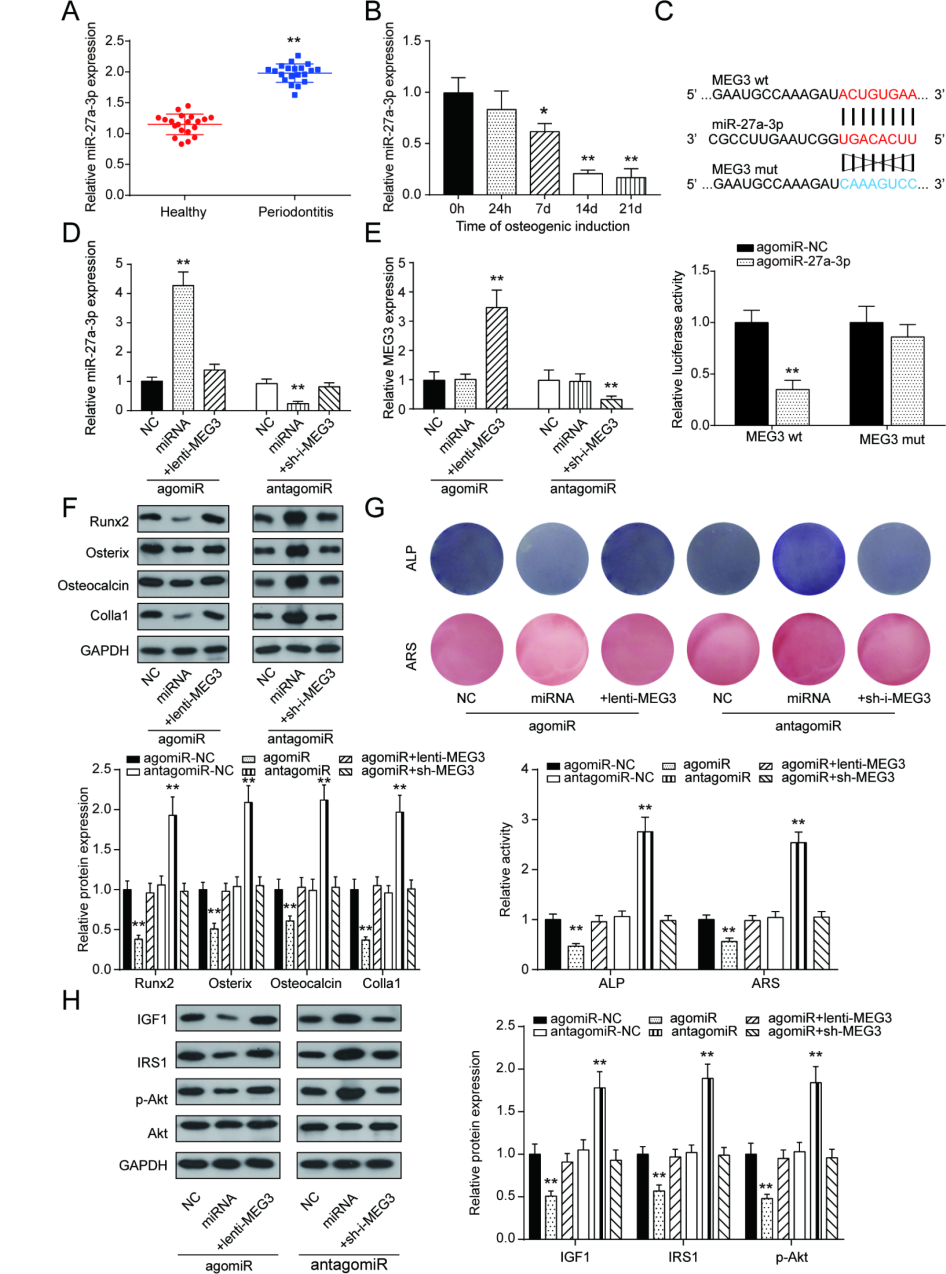
**LncRNA MEG3 regulated PDLSC osteogenic differentiation through miR-27a-3p.** (**A**) MiR-27a-3p expression in PDLSCs was up-regulated in periodontitis periodontal tissues compared with healthy periodontal tissues. (**B**) Expression of miR-27a-3p in 0h, 24h, 7d, 14d and 21d after osteogenic induction. (**C**) MiR-27a-3p directly targeted MEG3. Co-transfection of lncRNA MEG3-3’UTR-wt and agomiR-27a-3p significantly reduced relative luciferase activity. (**D**) Expression of miR-27a-3p in group of agomiR-27a-3p or antagomiR-27a-3p transfection (**E**) Expression of MEG3 in group of agomiR-27a-3p or antagomiR-27a-3p transfection. (**F**) Western blot of osteoblast makers including Runx2, Osterix, Osteocalcin and Colla1 in group of agomiR-27a-3p or antagomiR-27a-3p transfection. (**G**) ALP and Alizarin Red staining of osteogenic activities in the agomiR-27a-3p group and antagomiR-27a-3p group. (**H**) Protein expression of certain genes in PI3k/Akt signaling pathway, including IGF1, IRS1, p-Akt, and Akt. ** *P* < 0.01, compared with the NC group

Opposite to the qRT-PCR results of lncRNA MEG3 expression, miR-27a-3p expression in PDLSCs was up-regulated in periodontitis periodontal tissues compared with healthy periodontal tissues, and down-regulated after 14 days of osteogenic induction (Figure 4A, 4B). To further illustrate the targeting relationship between lncRNA MEG3 and miR-27a-3p, dual luciferase reporter gene assay was employed. Co-transfection of lncRNA MEG3-3’UTR-wt and agomiR-27a-3p significantly reduced relative luciferase activity, which confirmed that lncRNA MEG3 could directly target miR-27a-3p (Figure 4C, 4D). In group of agomiR-27a-3p transfection, miR-27a-3p expression was promoted, which could be neutralized by overexpression of lncRNA MEG3; in group of antagomiR-27a-3p transfection, miR-27a-3p expression declined, which could be weaken by silencing of lncRNA MEG3. In addition, MEG3 expression showed no significant change under down-regulation or up-regulation of miR-27a-3p (Figure 4E). Expression of Runx2, Osterix, Osteocalcin and Colla1 were down-regulated by agomiR-27a-3p, while up-regulated by antagomiR-27a-3p, indicating the negative correlation between miR-27a-3p and PDLSC osteogenic differentiation (Figure 4F). Subsequently, as shown by ALP and Alizarin Red staining, osteogenic activities could be suppressed in the agomiR-27a-3p group, and the suppression was relieved by lncRNA MEG3 overexpression; osteogenic activities boosted in the antagomiR-27a-3p group, and were reversed by silencing of lncRNA MEG3(Figure 4G). In addition, Figure 4H showed that with promotion of miR-27a-3p, protein expressions of certain genes in PI3k/Akt signaling pathway, including *IGF1*, *IRS1*, p-Akt, declined, which went the opposite way with suppression of miR-27a-3p. Taken together, these results indicated that miR-27a-3p negatively correlated with PDLSC osteogenic differentiation, as a target of lncRNA MEG3.

### MiR-27a-3p regulated PDLSC osteogenic differentiation through targeting *IGF1*

**Figure 5:**
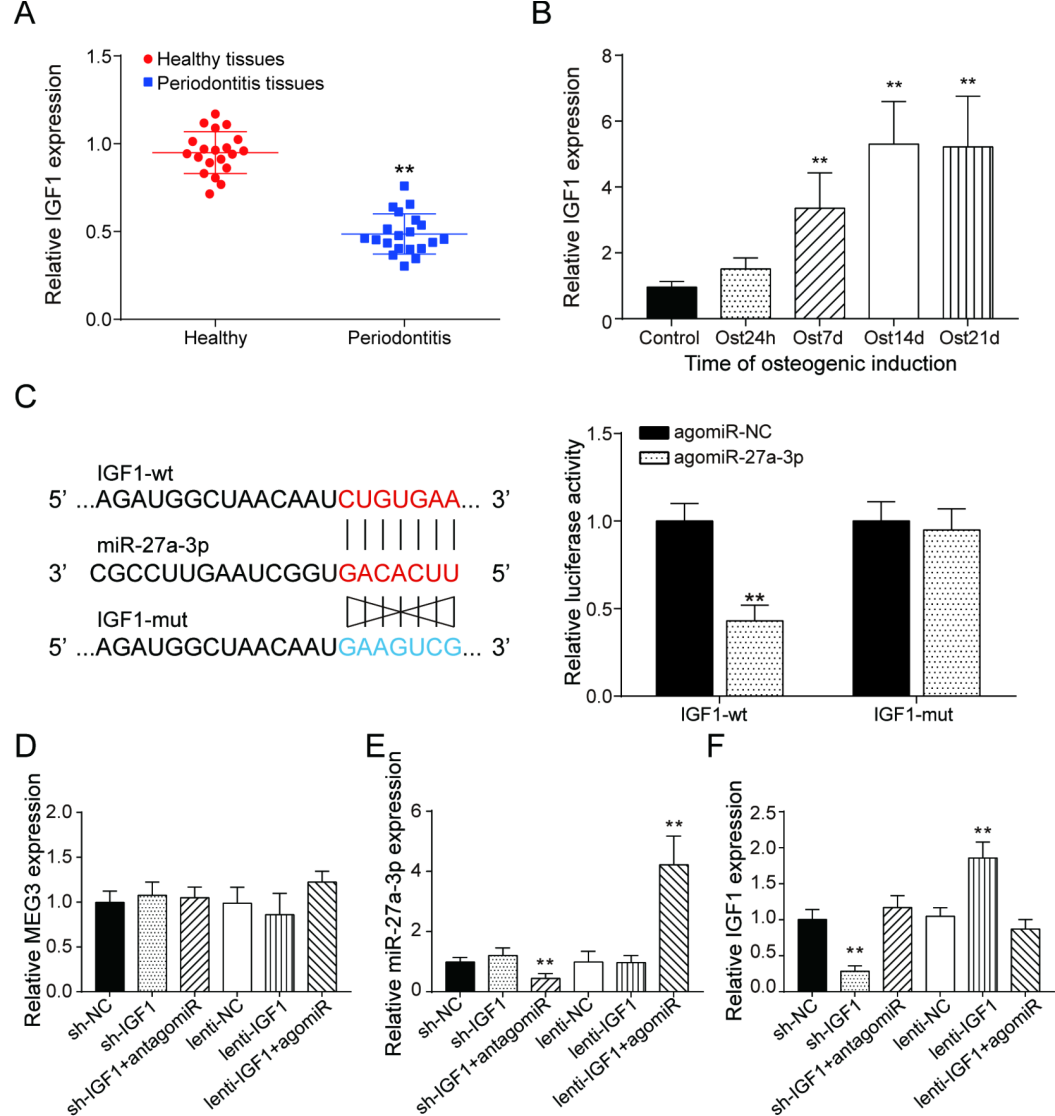
**LncRNA MEG3 was correlated to *IGF1* via MiR-27a-3p.** (**A**) *IGF1* expression in periodontitis periodontal tissues compared with healthy periodontal tissues. (**B**) Expression of *IGF1* in 0h, 24h, 7d, 14d and 21d after osteogenic induction. (**C**) MiR-27a-3p directly targeted *IGF1*. (**D**–**F**) Expression of MEG3, miR-27a-3p and *IGF1* in PDLSCs of each group. ** *P* < 0.01, compared with the NC group.

**Figure 6:**
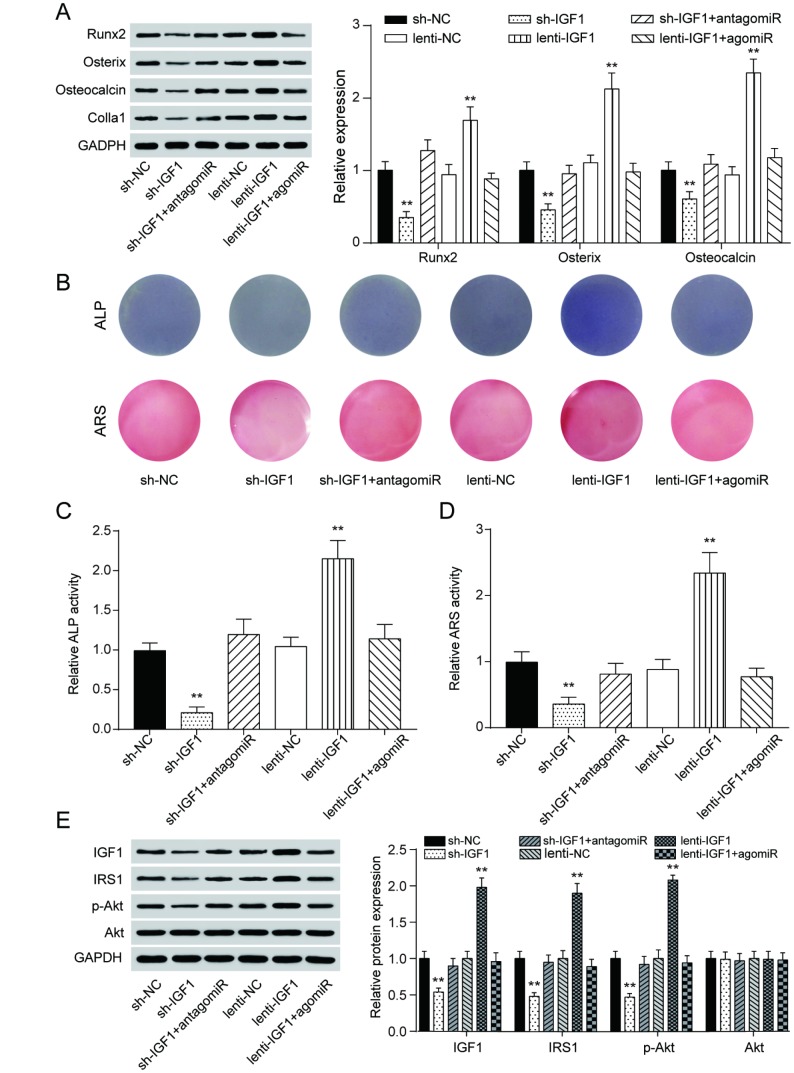
**MiR-27a-3p regulated PDLSC osteogenic differentiation through targeting IGF1.** (**A**) Western blot of Runx2, Osterix, Osteocalcin and Colla1 expression in PDLSCs with up-regulation or down-regulation of *IGF1*. (**B**–**D**) ALP and Alizarin Red staining of PDLSCs with different regulation of *IGF1*. (**E**) Protein expression of IGF1, IRS1, p-Akt and Akt in PDLSCs with different IGF1 regulation treatment. ** *P* < 0.01, compared with the NC group.

Similar to the expression of lncRNA MEG3, *IGF1* expression was lower in periodontitis periodontal tissues, compared with healthy periodontal tissues (Figure 5A). In older donors, expression of IGF1 were lower compared with the young donors (Supplementary Figure 3B). After 7 days of osteogenic induction, IGF expression in PDSLCs was tuned up (Figure 5B). Dual luciferase reporter gene assay confirmed the targeting relationship between miR-27a-3p and *IGF1* (Figure 5C). Deliberate suppression of *IGF1* expression by sh-IGF1 were neutralized by co-transfection of antagomiR-27a-3p; promotion of *IGF1* expression by lenti-IGF1 could be reversed by agomiR-27a-3p. However, little change in the expression of miR-27a-3p nor lncRNA MEG3 was shown under overexpression or suppression of *IGF1* (Figure 5D–5F). Western blot analysis showed that the inhibitory effects of agomiR-27a-3p on the expression of Runx2, Osterix, Osteocalcin and Colla1 could reverse the promoting effect by up-regulation of *IGF1*, while antagomiR-27a-3p could reverse the suppression of expressions of the three by sh-IGF1 (Figure 6A). Furthermore, ALP and Alizarin Red staining provided similar results that *IGF1* could counteract the inhibiting effect of miR-27a-3p on osteogenic activities (Figure 6B–6D). Besides, down-regulation of *IGF1* greatly suppressed relative protein expression of *IGF1*, *IRS1* and p-Akt (Figure 6E). In short, miR-27a-3p regulated PDLSC osteogenic differentiation through targeting *IGF1*.

### LncRNA MEG3/miR-27a-3p/*IGF1* axis regulated PI3K/Akt signaling pathway to affect PDLSC osteogenic differentiation

**Figure 7:**
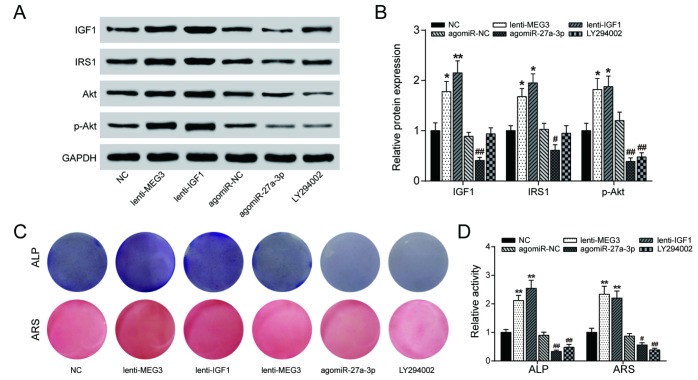
**LncRNA MEG3/miR-27a-3p/IGF1 axis regulated PI3K/Akt signaling pathway to affect PDLSC osteogenic differentiation.** (**A**, **B**) The results of western blot analysis indicated that overexpression of either lncRNA MEG3 or IGF1 promoted expression of PI3K/Akt-related proteins IRS1 and phosphorylated Akt (p-Akt), while agomiR-27a-3p and LY294002 blocked their expression. (**C**, **D**) Alizarin Red and ALP staining were also performed to validate this conclusion on cellular level. Increased calcium nodule formation and ALP activity were detected in lncRNA MEG3/IGF1 overexpression groups, while agomiR-27a-3p/LY294002 groups showed reverse results. ** *P* < 0.01, compared with the NC group. ## P＜0.01, compared with agomiR-NC group.

PI3K inhibitor LY294002 (10 nM) was used to explore the effects of lncRNA MEG3/miR-27a-3p/*IGF1* axis on the PI3K/Akt signaling pathway. The results of western blot analysis indicated that overexpression of either lncRNA MEG3 or *IGF1* promoted expression of PI3K/Akt-related proteins *IRS1* and p-Akt, while agomiR-27a-3p and LY294002 did the opposite (Figure 7A, 7B), indicating that lncRNA MEG3 might sponge miR-27a-3p for up-regulation of *IGF1*, leading to the activation of PI3K/Akt signaling pathway. Alizarin Red and ALP staining results validated this conclusion on a cellular level – osteogenic activities increased with lncRNA MEG3 or *IGF1* upregulated, and declined under effect of agomiR-27a-3p or LY294002 (Figure 7C, 7D). To conclude, lncRNA MEG3/miR-27a-3p/*IGF1* axis regulated PI3K/Akt signaling pathway to affect PDLSC osteogenic differentiation.

## DISCUSSION

To sum up, the expression levels of lncRNAMEG3 and *IGF1* were both down-regulated, while miR-27a-3p was up-regulated in periodontitis periodontal tissues compared with healthy periodontal tissues. Furthermore, both lncRNA MEG3 and *IGF1* could be directly targeted by miR-27a-3p. In addition, lncRNA MEG3 sponged miR-27a-3p for up-regulation of *IGF1* to result in activation of PI3K/Akt signaling pathway. Taken together, lncRNA MEG3/miR-27a-3p/*IGF1* axis played a role in PDLSC osteogenesis differentiation via activating PI3K/Akt signaling pathway.

Bioinformatic analyses have greatly improved the ability of researchers in processing and analyzing biological data. The expression of the targets such as DNA [21], RNA [22] and signaling pathway [23] could be directly observed through microarray and gene network analysis to assess candidate mechanisms. Herein, gene set enrichment analysis (GSEA) was performed and a co-expression network of lncRNA/miRNA/mRNA was conducted for further annotation, visualization, and integrated discovery. The results that lncRNA influenced miRNA and miRNA influences mRNA subsequently, which were consistent with most of previous studies [24, 25].

Many recent researches have investigated human diseases through lncRNAs, leading to a new way to find the internal mechanism for better prognoses and treatments. However, few researchers aimed at studying lncRNAs in periodontitis. Most of the studies were related to the intrinsic mechanism of lncRNA from the perspective of periodontitis, such as chronic periodontitis (CP) [26] and aggressive periodontitis (AgP) [27], and seldom of them focused on PDLSCs. Besides, most of researches focusing on lncRNA MEG3 showed that lncRNA MEG3 involved in multifarious cancers was down-regulated in most cancers and affected cell proliferation, progression, and prognosis [28, 29] which indicated there was still a large research space on the influence mechanism of lncRNA MEG3 in periodontitis PDLSCs and our study was of great practical significance.

Furthermore, lncRNA/miRNA/mRNA regulatory network that lncRNA functioned as ceRNA to impact miRNAs at posttranscriptional processing steps has been widely identified, thus leading to inhibition of miRNA target genes recently [25]. For example, lncRNA POIR was found to form a regulatory network with miRNA-182 and *FoxO1*, which taken a notable up-regulated effect on periodontitis PDLSCs osteogenic differentiation [20] and miR-147 was down-regulated by up-regulation of lncRNA MEG3 to result in increased expression of *Sox2,* which activated NF-kB pathway and Wnt/β-catenin pathway in PC12 cells after hypoxia [30]. Besides, Galani V *et al*. discovered that lncRNA MEG3 genes and IGF signaling family genes included IGF1 are both associated with meningioma progression [31] which led to a guess that endogenous cross-talk maybe exist between lncRNA MEG3 and *IGF1* in PDLSCs. Moreover, Yu Y *et al*. found that *IGF1* can facilitate the osteogenic differentiation and osteogenesis of human PDLSCs via ERK and JNK MAPK pathway, suggesting that IGF1 was a potent endogenous agent for PDLSCs regeneration [32]. Based on these findings, we speculated that lncRNA MEG3 may participate in this system of periodontitis PDLSCs and act as a ceRNA, and searched for potential interactions between lncRNA MEG3 and *IGF1*. As expected, we found that lncRNA MEG3 could sponge miR-27a-3p, resulting in *IGF1* up-regulation, and PI3K/Akt signaling pathway was activated, all which promoted PDLSC osteogenic differentiation ultimately.

Altogether, the findings presented in this study highlights that lncRNA MEG3 was determined as a biomarker as periodontitis inhibitor because of the new-found mechanism underlying lncRNA MEG3/miR-27a-3p/*IGF1* axis via PI3K/Akt signaling pathway by promoting osteogenic differentiation in PDLSCs. Moreover, our result showed that lncRNA MEG3/miR-27a-3p/*IGF1* axis definitely played a significant role in understanding the pathogenesis of periodontitis for better prognosis. However, because of the limitation of the samples we researched in this study, further confirmation should be needed in prospective researches of the pathological and physiological mechanism according to lncRNA MEG3/miR-27a-3p/*IGF1* axis in periodontitis PDLSCs. Moreover, effects of MEG3/miR-27a-3p/*IGF1* on osteoclast should also be investigated in further studies.

To conclude, lncRNA MEG3 sponged miR-27a-3p to up-regulate *IGF1* to activate PI3k/Akt signaling pathway to promote PDLSC osteogenic differentiation in periodontitis.

## MATERIALS AND METHODS

### Bioinformatic analysis

Microarray data of both lncRNA and mRNA expression profiles of 3 periodontitis periodontal tissues and 3 healthy periodontal tissues were downloaded from Gene Expression Omnibus database (GEO) (https://www.ncbi.nlm.nih.gov/geo/). The series accession number of microarray data in this study was GSE78074 and matched platform was GPL16976. Differentially expressed lncRNAs and mRNAs between periodontitis periodontal tissues and healthy periodontal tissues were selected out and visualized using R software (version 3.4.1) “limma” package. In brief, data was processed for selecting out differentially expressed genes (DEGs) with screening criteria adj *P* value <0.05 and |FC (Fold Change)|>2. In addition, Gene Set Enrichment Analysis (GSEA) software (http://software.broadinstitute.org/gsea) and kyoto Encyclopedia of Genes and Genomes (KEGG) database (https://www.genome.jp/kegg/) were adopted to unveil significantly enriched signaling pathways. Top enriched signaling pathways (adjusted *P*<0.01) from GSEA results were visualized by dotplot and joyplot using R software “ggplot2” and “easygplot2” packages. Cytoscape (version 3.6.0) was used to conduct co-expression network analysis based on differentially expressed lncRNAs and mRNAs. Node and edge files that revealed the coefficient factor threshold>0.7 and adjusted *P*<0.05 were produced using R project. Signaling pathway-related miRNAs or mRNAs were predicted using DIANA Tools (http://diana.imis.athena-innovation.gr/DianaTools) or string (https://string-db.org/) respectively.

### Clinical tissues

Human periodontitis PDLSCs (pPDLSCs) were obtained from periodontal ligament tissues of 20 periodontitis patients (10 males, 10 females, age 24-38), who received premolar extraction at stomatological hospital of Shandong University. Human healthy PDLSCs (hPDLSCs) were obtained from periodontal ligament tissues of premolars extracted for orthodontic treatment from 20 healthy individuals (10 males, 10 females, age 18-25). All procedures in the study were scrutinized and ratified by Shandong University & Department of Orthodontics Committee of stomatological hospital of Shandong University and tissue donors in the study signed informed consent allowing the use of their tissues for scientific researches.

### Cell culture

Premolars were firstly disinfected with 75% ethanol and washed by PBS. Normal or periodontitis periodontal ligament tissues were then separated from middle 1/3 of teeth roots. Separated tissues were shred and digested using collagenase type I (1 mg/mL; Sigma-Aldrich, MO, USA) for 15 min at 37°C. Cell suspensions were then filtered and cultured in low-glucose DMEM (Gibco, Carlsbad, CA, USA) with 10% fetal bovine serum (FBS, Gibco, Carlsbad, CA, USA) at 37°C in a humidified atmosphere of 5% CO_2_. After 2-week incubation, cells were digested and single cell-derived PDLSC colony cultures were obtained by limiting dilution. PDLSCs were collected and utilized for following experiments after 5 times of passages. Human embryo kidney cell line HEK-293 used for dual-luciferase assay was purchased from BeNa Culture Collection (Beijing, China) and cultured in high-glucose DMEM (Gibco, Carlsbad, CA, USA) with 10 % FBS.

### Flow cytometry analysis

A total of 1×10^6^ PDLSCs was incubated with the antibodies against CD90, CD105, CD146, CD45, CD34, CD11b, CD19, STRO-1 and HLADR according to MSC characterization guidelines [33]. The isotype IgG1 antibodies were applied as controls (ab154450, 0.1 μg, Abcam). Thereafter, flow cytometry was operated on FACSCanto^TM^ II Flow Cytometer (BD Biosciences, CA, USA). μμμμμ

### Cell transfection

AgomiR-27a-3p, antagomiR-27a-3p, lentiviral-pEF-1a/Puro-MEG3 (lenti-MEG3), lentiviral-pGLVU6/Puro-sh-MEG3 (sh-MEG3), lentiviral-pEF-1a/Puro-IGF1 (lenti-IGF1), lentiviral-pGLVU6/Puro-sh-IGF1 (sh-IGF1) and their corresponding negative controls (NC) in this study were all bought from GenePharma (Shanghai, China). Before transfection, 2 × 10^5^ cells in logarithmic growth phase were plated into six-well plates, with 2 mL complete medium for 16-18 h, until they were 30-40% confluent. AgomiR-27a-3p and antagomiR-27a-3p were transfected into pPDLSCs using Lipofectamine 3000 reagent (Invitrogen, Carlsbad, CA, USA), with all procedures following the manufacturer’s protocol. The transfection concentration was 60 nM. For lentivirus transfection of the PDLSCs, the supernatant cell culture medium was replaced with fresh cell culture medium containing 6 μg/mL polybrene and lentivirus particles (MOI=20). Then the cells were incubated for 24 h, before the medium was replaced with fresh medium. Stable transfected cells were filtered with puromycin. For LY294002 treatment, the pPDLSCs were cultured with 10 nM LY294002 (Beyotime) according to a previous study [34].

### Osteoblastic induction

Osteoblastic induction was performed using StemPro^TM^ Osteogenesis Differentiation Kit (Gibco, Carlsbad, CA, USA). In brief, hPDLSCs were washed with PBS and seeded onto 12-well plates, then cultured with MSC Growth Medium at 37°C in a humidified atmosphere of 5% CO_2_ for 5 days. Afterwards, culture medium was replaced by Complete Osteogenesis Differentiation Medium (Invitrogen, Carlsbad, CA, USA) and PDLSCs would differentiate to osteoblasts.

### Quantitative real-time PCR (qRT-PCR)

Total RNA of PDLSCs was extracted with TRIzol reagent (Invitrogen). Thereafter, about 2 μg of total RNA was reversely transcripted to cDNA using SuperScript^TM^ III Reverse Transcriptase Kit (Invitrogen) for lncRNA/mRNA reverse transcription and miRCURY LNA RT Kit (Qiagen, Duesseldorf, Germany) for miRNA reverse transcription. Subsequently, qRT-PCR was then performed using SYBR Green qPCR Master Mix (Takara, Tokyo, Japan) on LightCycler 480 PCR System (Roche, Rotkreuz, Switzerland). Normalization of mRNA and lncRNA expression values was performed against GAPDH and miRNA expression value was normalized against U6. Relative RNA expression was calculated with 2^-ΔΔCt^ method. PCR primers were synthesized by Sangon Biotech (Shanghai, China) and primer sequences were shown in Table 1. Working concentration of primers used for qRT-PCR in this study was 0.5 μmol/L.

**Table 1:** Primer sequences for qRT-PCR

**Gene**	**Forward primer 5’-3’**	**Reverse primer 5’-3’**
MEG3	TACACCTCACGAGGGCACTA	CAGGGCTTAATGCCCAATGC
miR-27a-3p	GTTCACAGTGGCTAAGTTCCGC	Involved in the kit
IGF1	ATAGAGCCTGCGCAATGGAA	ACTGAAGAGCATCCACCAGC
GAPDH	CCGCATCTTCTTTTGCGTCG	GGACTCCACGACGTACTCAG

### Western blot analysis

Total protein from PDLSCs (5×10^5^ cells) was extracted 24h after transfection using RIPA lysis buffer (Beyotime, Shanghai, China) and quantified using Enhanced BCA Protein Assay Kit (Beyotime). 20 μg of total protein was then separated by SDS-PAGE and transferred onto PVDF membranes (Beyotime). 5% bovine serum albumin (BSA, Sigma-Aldrich) was used as a block for 30 min at room temperature, causing incubation of the membranes with primary antibodies overnight at 4°C (GAPDH was used for internal reference). Secondary antibody was then added and incubated for another 1 h at room temperature. HRP-labeled proteins were developed using BeyoECL Star Kit (Beyotime) and filmed after washed by TBST for 3 times. Primary antibodies were: rabbit anti-IGF1 (ab9572, 1:2000, Abcam), rabbit anti-IRS1 (ab40777, 1:2000), rabbit anti-pan-Akt (ab8805, 1:500), rabbit anti-pan-Akt (phospho T308; ab38449, 1:500), rabbit anti-Runx2 (ab23981, 1:1000), rabbit anti-Osterix (ab22552, 1:2000), rabbit anti-Osteocalcin (ab93876, 1:500), rabbit anti-type-1 collagen (Colla1, ab34710, 1:2000) and rabbit anti-GAPDH (ab181602, 1:10000). Secondary antibody was HRP labeled goat anti-rabbit IgG (ab6721, 1:2000). Antibodies in this study were purchased from Abcam (MA, USA). Proteins were visualized by ECL-plus reagents (Millipore, Billerica, MA, USA) and Image J software (Version1.48u, Bethesda, USA) was employed for the measurement of band density.

### Dual luciferase reporter gene assay

Binding sites between miRNAs and lncRNAs were predicted using miRcode (http://www.mircode.org/) and targeted relationships between miRNAs and mRNAs were predicted using TargetScan 7.1 (http://www.targetscan.org/vert_71/). PmirGLO vectors (Promega, Madison, WI, USA) containing wild-type (wt) / mutation-type (mut) sequences of MEG3 or *IGF1* 3’-untranslated region (3’-UTR) were constructed using XL Site-directed Mutagenesis Kit (Qiagen). PmirGLO luciferase reporter gene vector loaded with MEG3-3’UTR-wt or MEG3-3’UTR-mut was introduced into HEK-293 cells with co-transfection of AgomiR-203b-3p or AgomiR-NC using Lipofectamine 3000 reagent (Invitrogen, Carksbad, CA, USA). Similarly, luciferase reporter gene vector loaded with *IGF1*-3’UTR-wt or *IGF1*-3’UTR-mut was introduced into HEK-293 cells with co-transfection of AgomiR-203b-3p or AgomiR-NC by using Lipofectamine 3000 reagent. Relative luciferase activity was assessed with Dual-luciferase Reporter Assay Kit (Promega) following the manufacturer’s protocol 24 h after transfection.

### Alkaline Phosphatase (ALP) staining

ALP staining was performed using BCIP/NBT Alkaline Phosphatase Color Development Kit (Beyotime) at 14^th^ day after osteoblastic induction. Cells cultured in 12-well plates were rinsed and fixed with PBS for 3 times and 4% paraformaldehyde for 30 min successively. Subsequently, BCIP/NBT ALP staining buffer was added and incubated for 2 h at room temperature in a dark room. Then reaction was terminated and the ALP activity was quantified by spectrophotometric absorbance at 405 nm.

### Alizarin Red staining (ARS)

Alizarin red staining was performed at 21^st^ day after osteoblastic induction. Cells cultured in 12-well plates were rinsed with Phosphate Buffered Saline (PBS) buffer (pH 7.4) for 3 times and fixed with 4% paraformaldehyde for 30 min. Afterwards, cells were stained with 1% alizarin red S solution (pH=8.4) (Sigma-Aldrich) for 5 min. Thereafter, cells were washed with PBS for 3 times and observed under a light microscope. To quantify mineralization, bound dye was extracted in 10 mM sodium phosphate containing 10% cetylpyridinium chloride and quantified spectrophotometrically at 562 nm.

### Statistical analysis

All the experiments were repeated test at least thrice. Data was analyzed with GraphPad Prism version 6.0 (GraphPad Software, CA, USA) and presented as mean value ± standard deviation (mean ± SD) in this study. Multiple comparisons between two groups were implemented by student’s *t* test, and differences between three or more groups were evaluated by one-way ANOVA. *P* value less than 0.05 was deemed statistically significant.

### Ethical statement

Investigation has been conducted in accordance with the ethical standards and according to the Declaration of Helsinki and according to national and international guidelines and has been approved by Shandong University & Department of Orthodontics committee.

## Supplementary Material

Supplementary Figures
