# Supplementary material for: Down-regulation of long non-coding RNA MEG3 suppresses osteogenic differentiation of periodontal ligament stem cells (PDLSCs) through miR-27a-3p/IGF1 axis in periodontitis
Source: Aging (Albany NY). 2019 Aug 9;11(15):5334–50. doi: 10.18632/aging.102105 (PMC6710065; doi:10.18632/aging.102105)
Supplement: Supplementary Figures [file aging-11-102105-s001.pdf]

SUPPLEMENTARY FIGURES

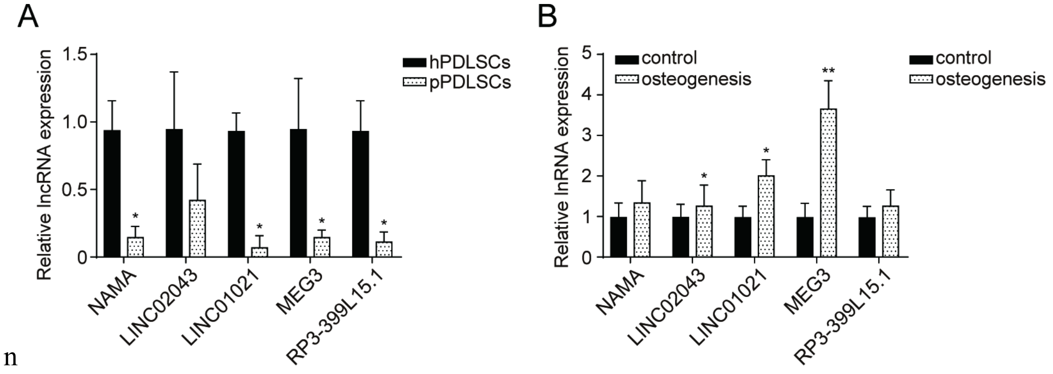

n

Supplementary Figure 1. QRT-PCR of dys-regulated lncRNAs in healthy and periodontitis PDLSCs. (**A**) Expression of NAMA, LINC02043, LINC01021, MEG3 and RP3-399L15.1 was down-regulated in periodontitis PDLSCs compared with that in healthy samples, and thereinto MEG3 displayed the most remarkable difference. (**B**) After osteogenesis treatment, down-regulation of LINC02043, LINC01021, MEG3 was significantly reversed and thereinto the increase of MEG3 was the most distinct. * $P < 0.05$, ** $P < 0.01$, compared with the hPDLSCs or control group

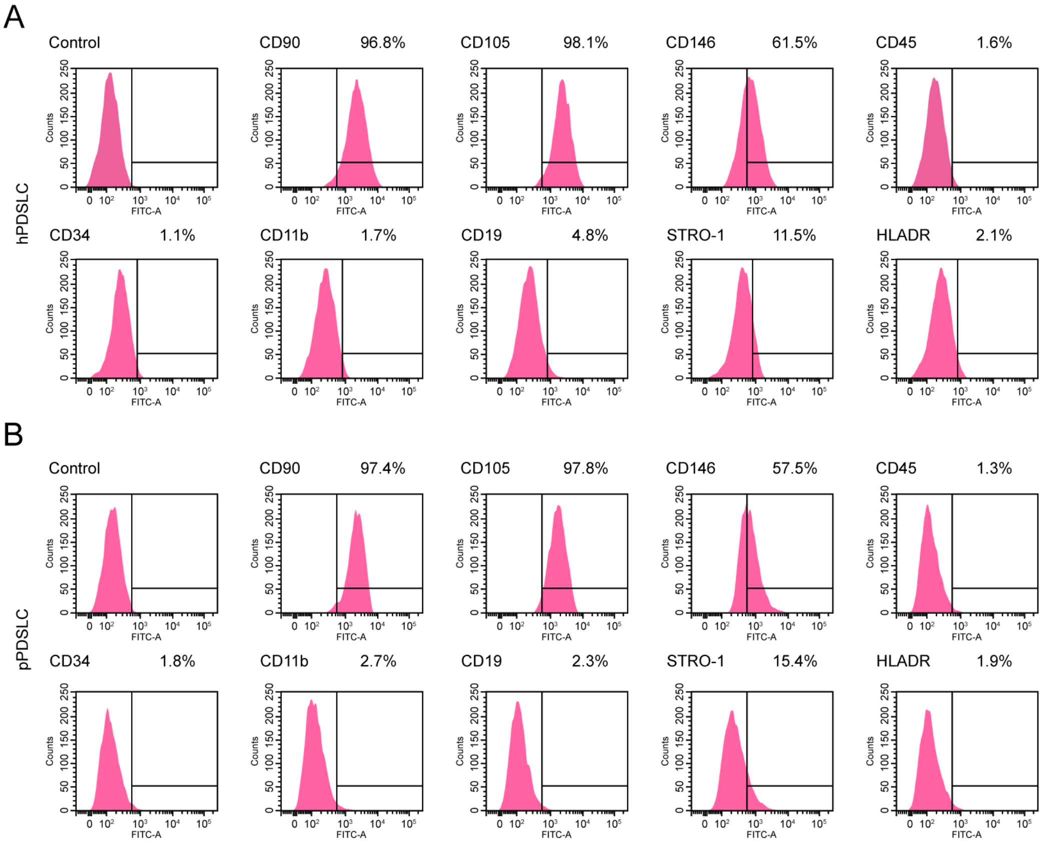

Supplementary Figure 2. Detection of PDLSC surface antigens by flow cytometry. (**A**) Expression of CD90, CD105, CD146, CD45, CD34, CD11b, CD19, STRO-1, and HLADR in hPDLSCs. (**B**) Expression of CD90, CD105, CD146, CD45, CD34, CD11b, CD19, STRO-1, and HLADR in pPDLSCs.

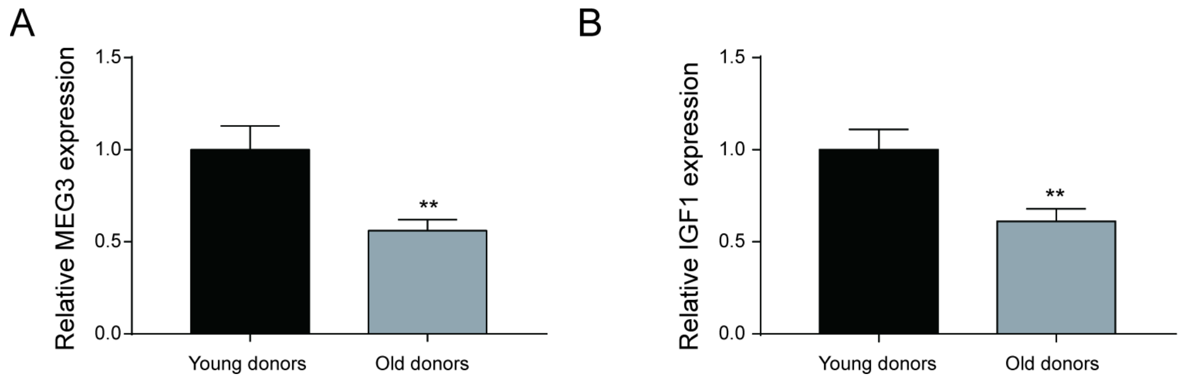

Supplementary Figure 3. Detection of MEG3 and IGF1 in pPDLSCs from old donors and young donors. (**A**) Expression of MEG3 was lower in the old donors (**B**) Expression of IGF1 was lower in the old donors. older donor: age > 60, number = 10; young donor: age between 24-38, number = 20. ** $P < 0.01$
